# Supplementary material for: Nerve injury-induced protein 2 preserves lysosomal membrane integrity to suppress ferroptosis
Source: eLife. 2026 Sep 11;15:RP110919. doi: 10.7554/eLife.110919 (PMC13568700; doi:10.7554/eLife.110919)
Supplement: Supplementary file 4 [file elife-110919-fig2-data1.zip › Figure 2-Source data 1.pdf]

Figure 2B

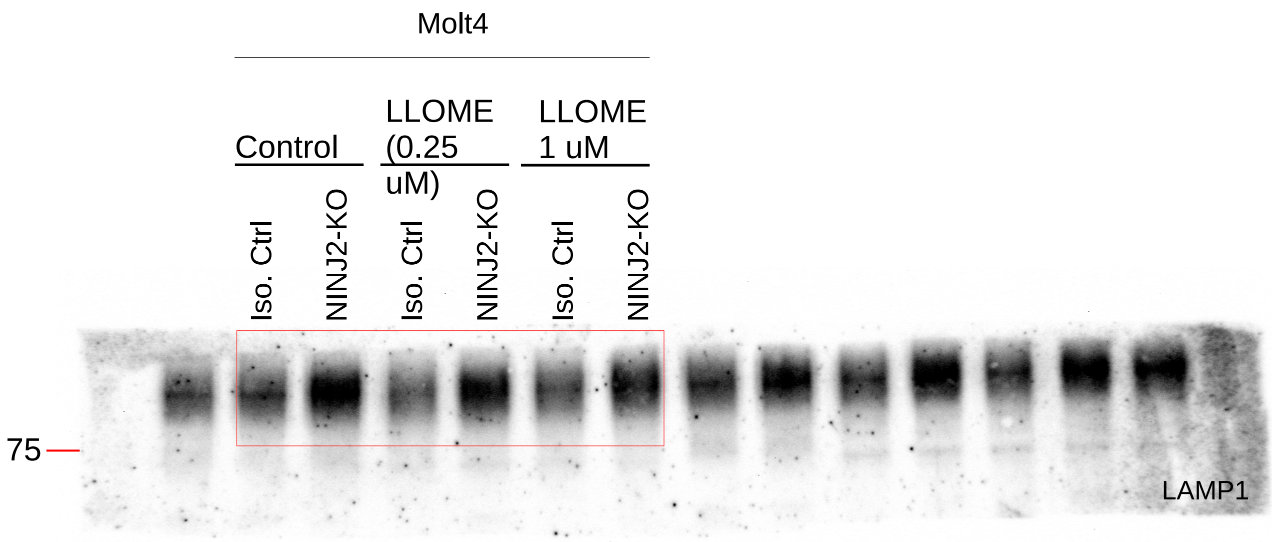

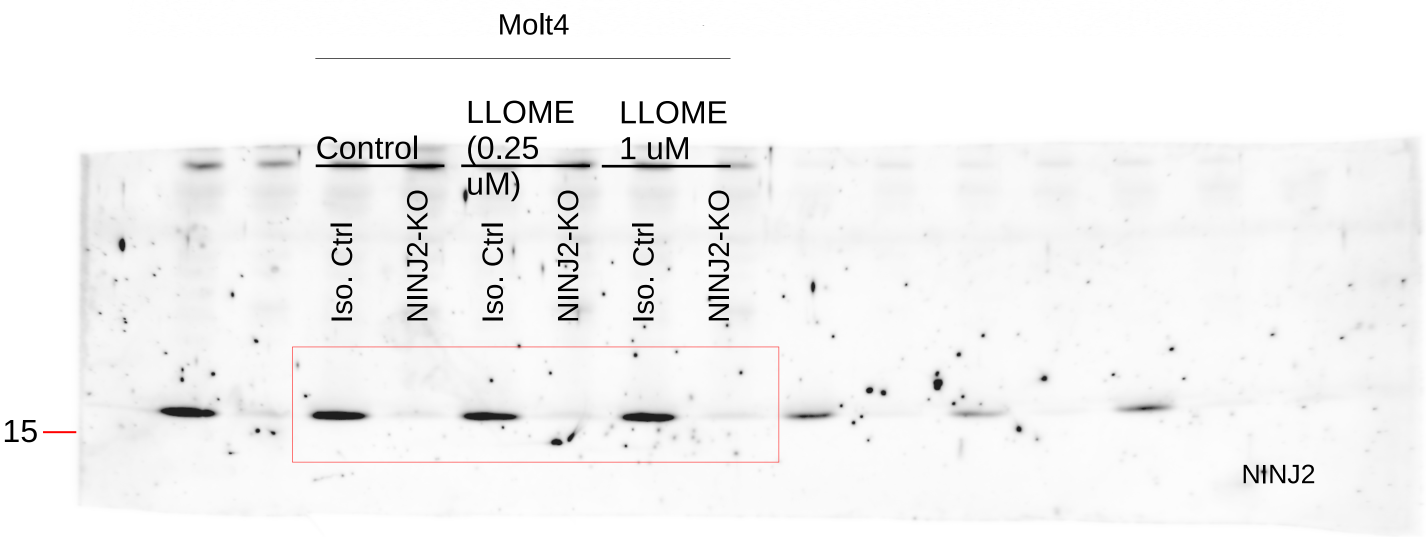

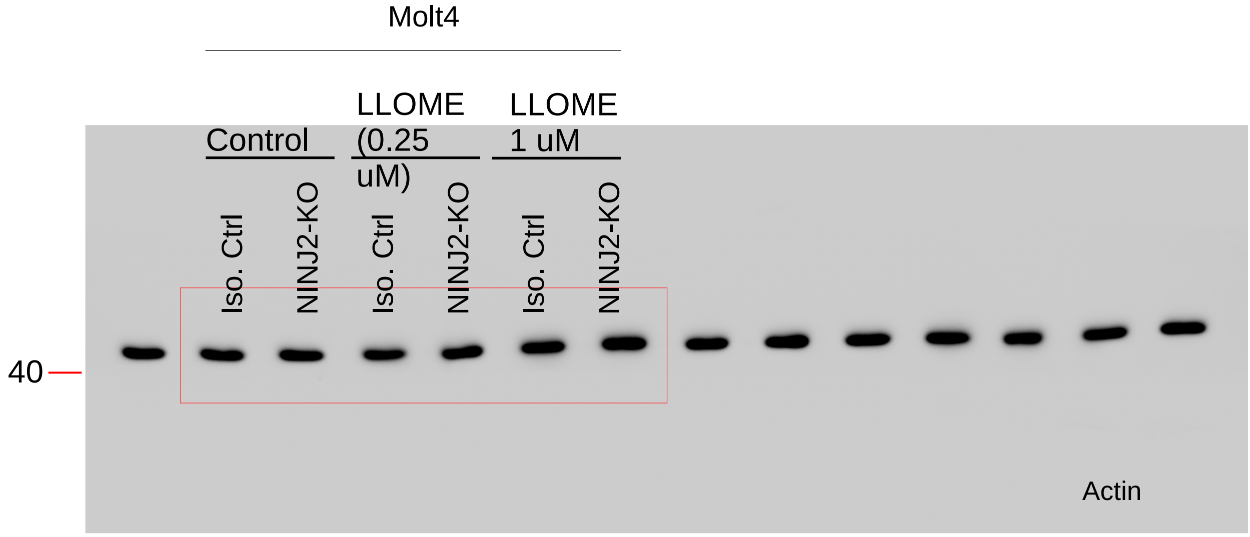

Figure 2, Source Data 1. Original membranes corresponding to Figure 2 panel B.

Figure 2C

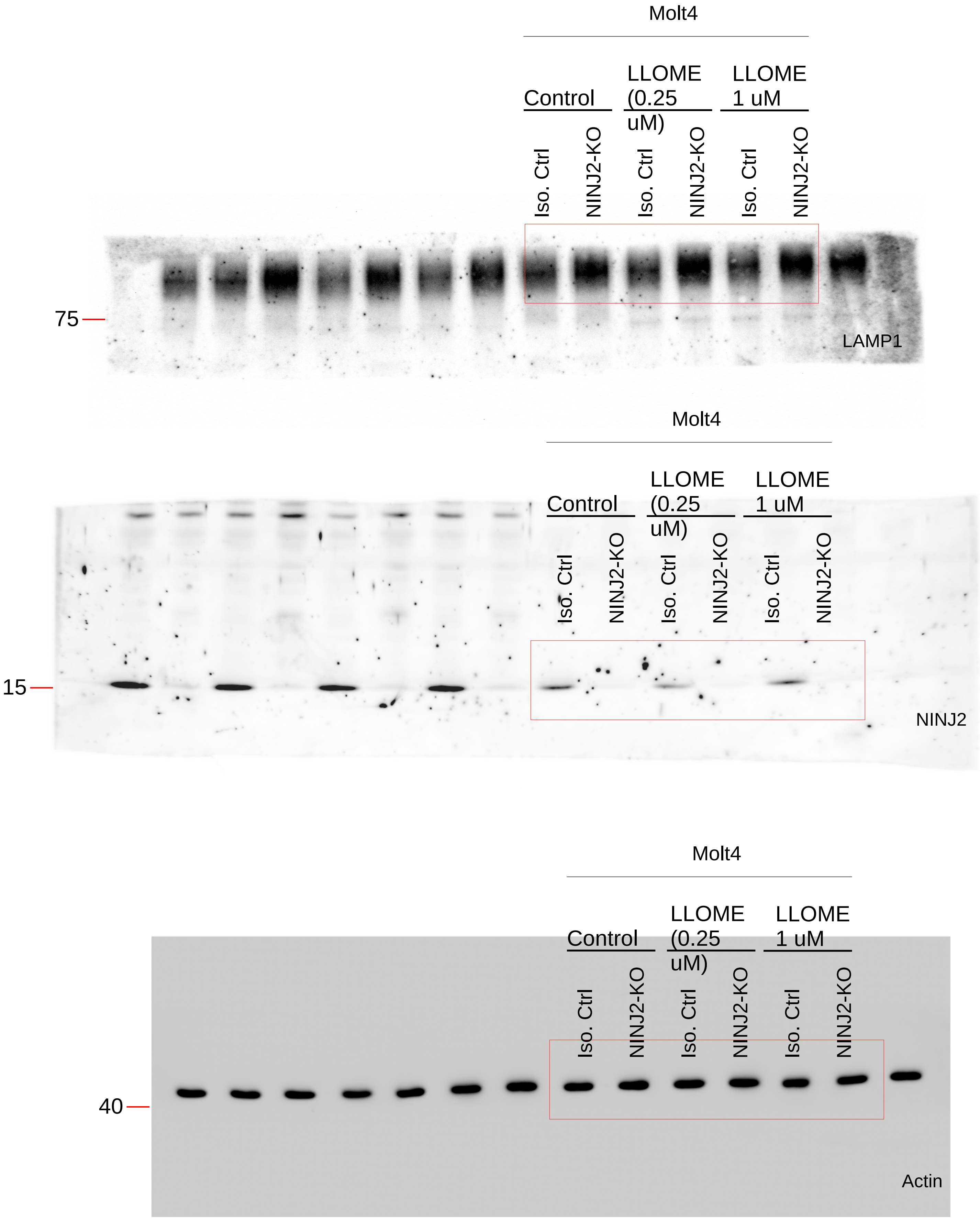

Figure 2, Source Data 1. Original membranes corresponding to Figure 2 panel C.

Figure 2D

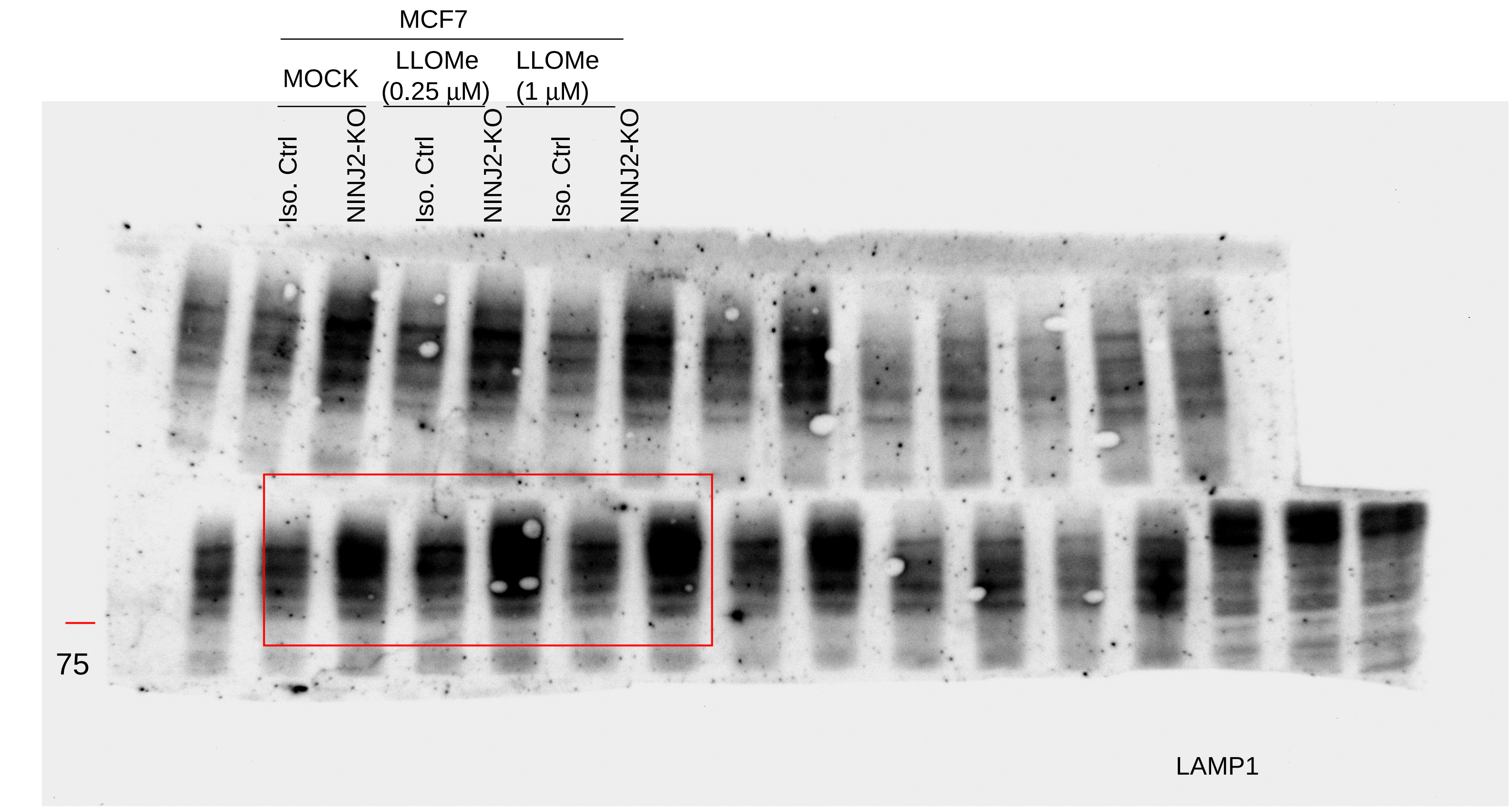

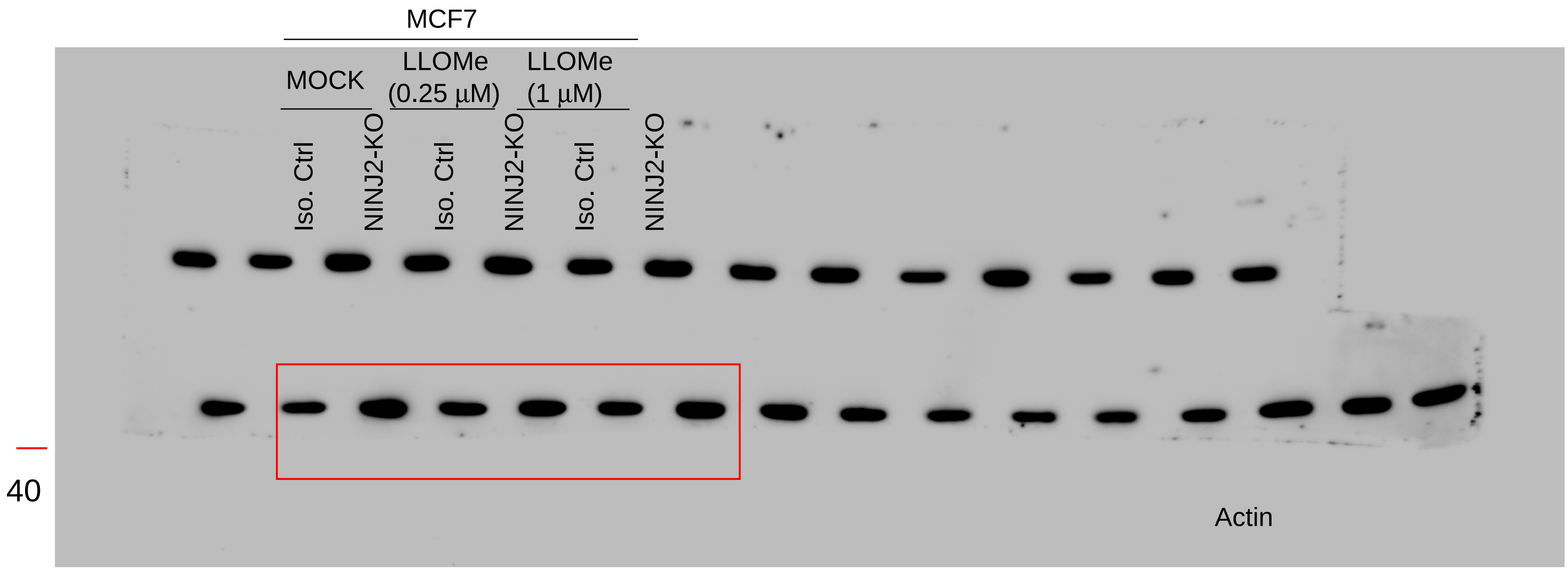

Figure 2, Source Data 1. Original membranes corresponding to Figure 2 panel D.

Figure 2E

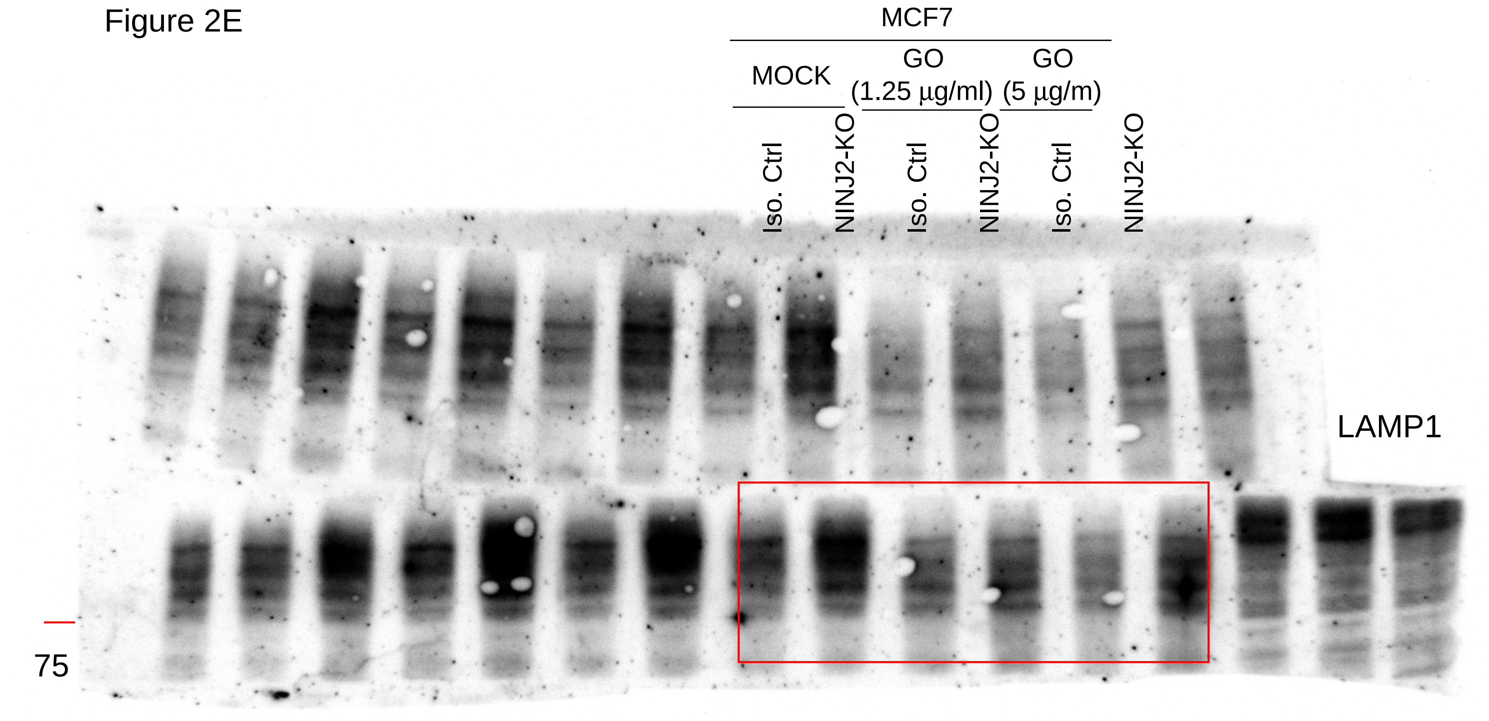

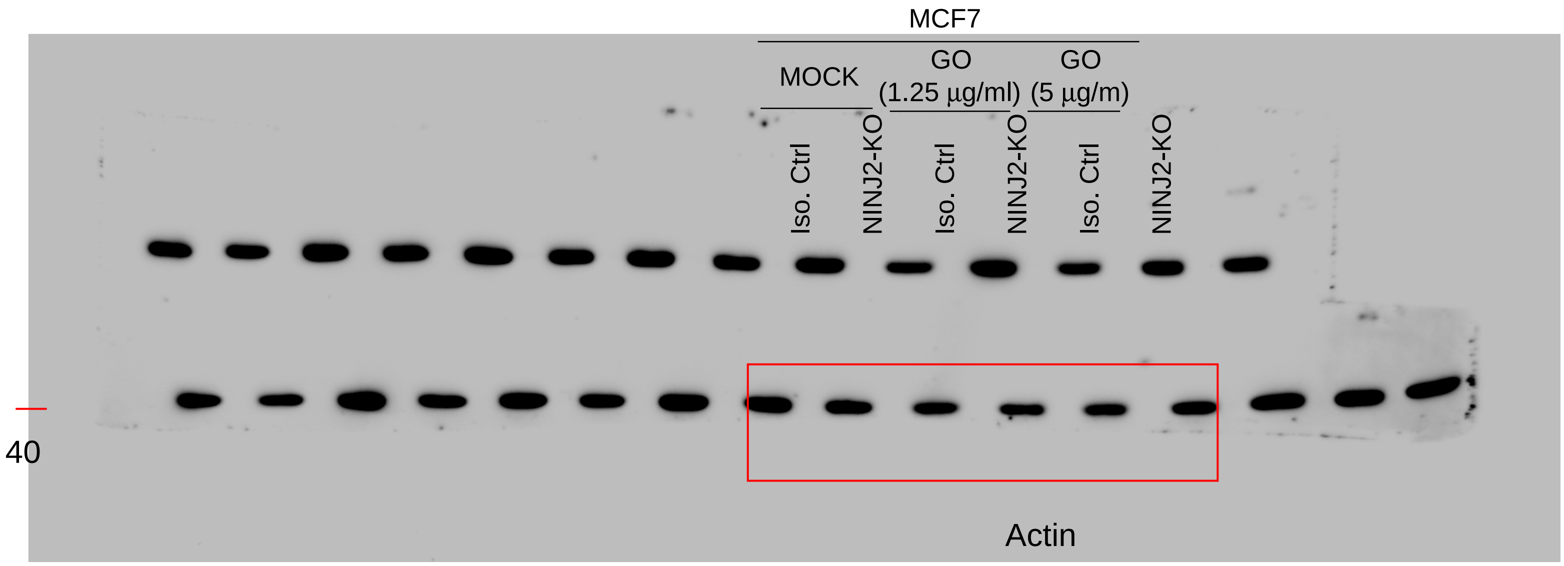

Figure 2, Source Data 1. Original membranes corresponding to Figure 2 panel E.
