# Supplementary material for: Nerve injury-induced protein 2 preserves lysosomal membrane integrity to suppress ferroptosis
Source: eLife. 2026 Sep 11;15:RP110919. doi: 10.7554/eLife.110919 (PMC13568700; doi:10.7554/eLife.110919)
Supplement: Supplementary file 8 [file elife-110919-fig3-data1.zip › Figure 3-Source data 1.pdf]

Figure 3C

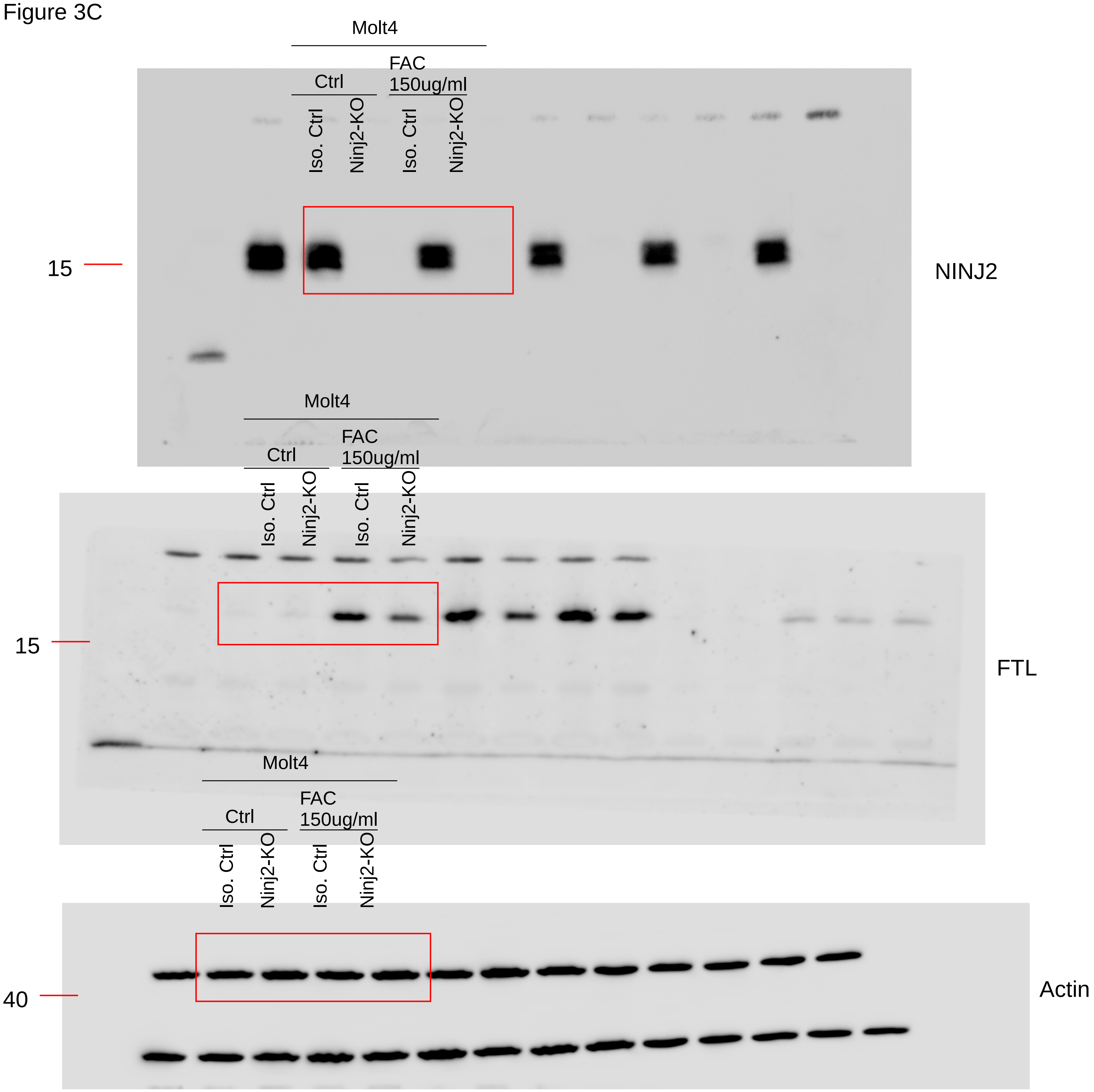

Figure 3, Source Data 1. Original membranes corresponding to Figure 3 panel C.

Figure 3D

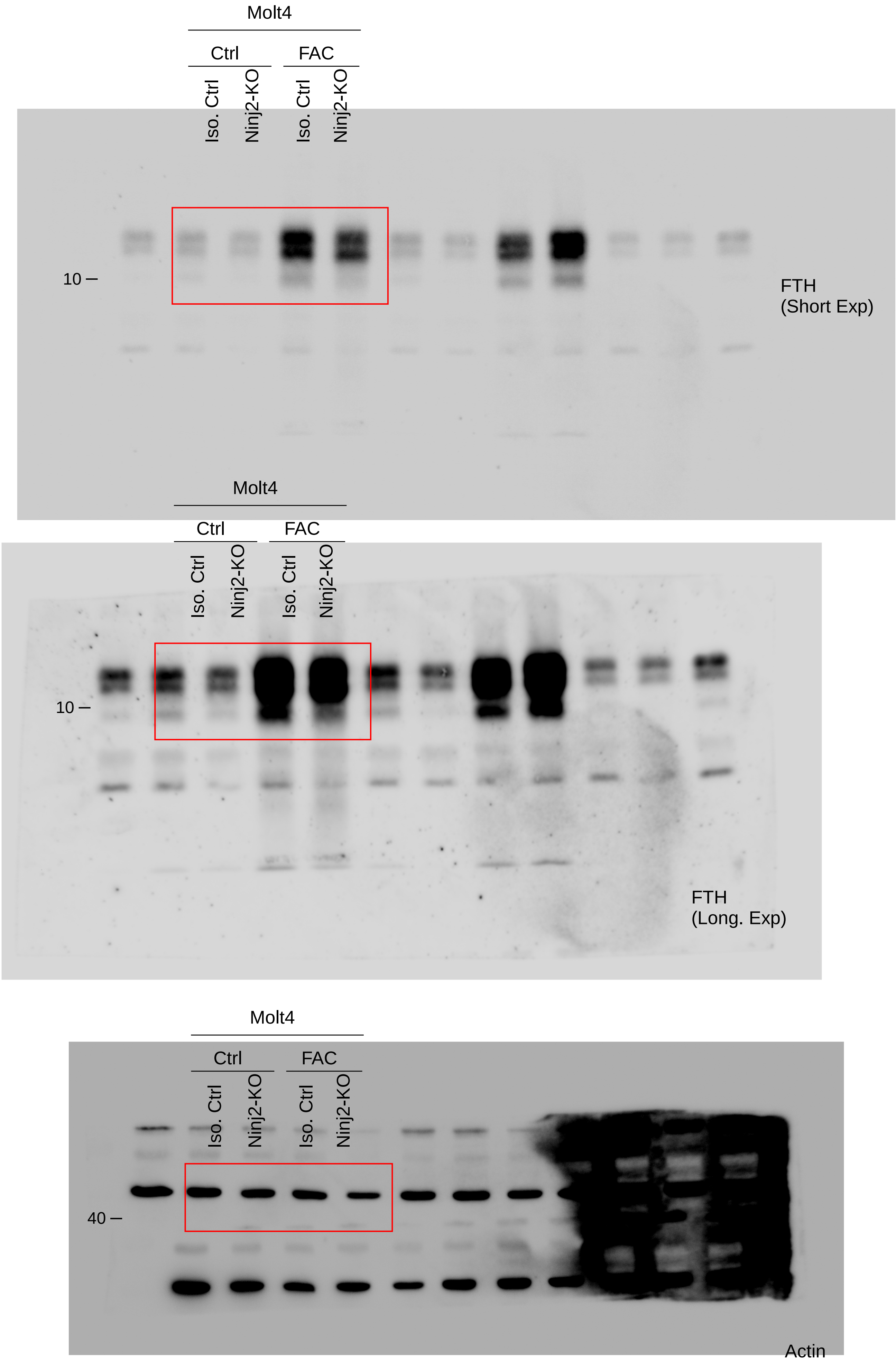

Figure 3, Source Data 1. Original membranes corresponding to Figure 3 panel D.

Figure 3E

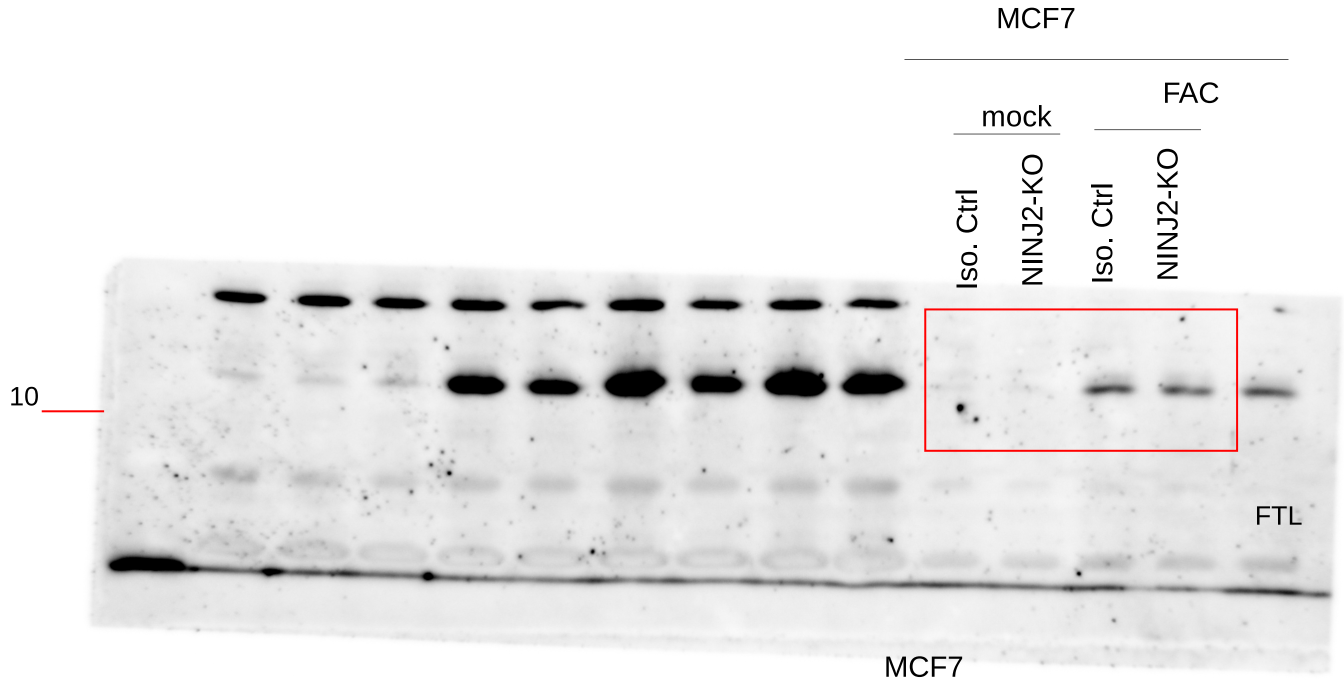

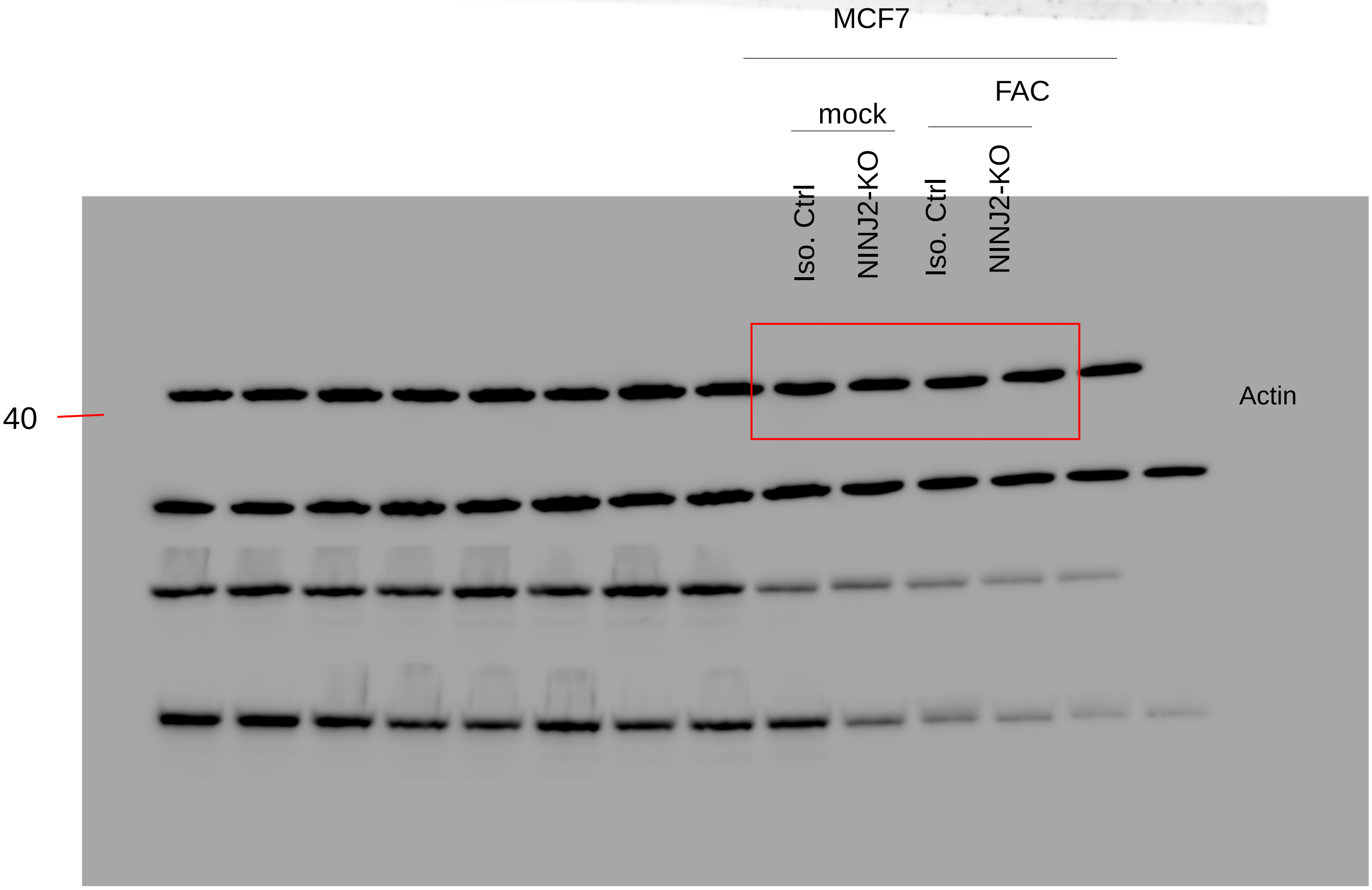

Figure 3, Source Data 1. Original membranes corresponding to Figure 3 panel E.

Figure 3F

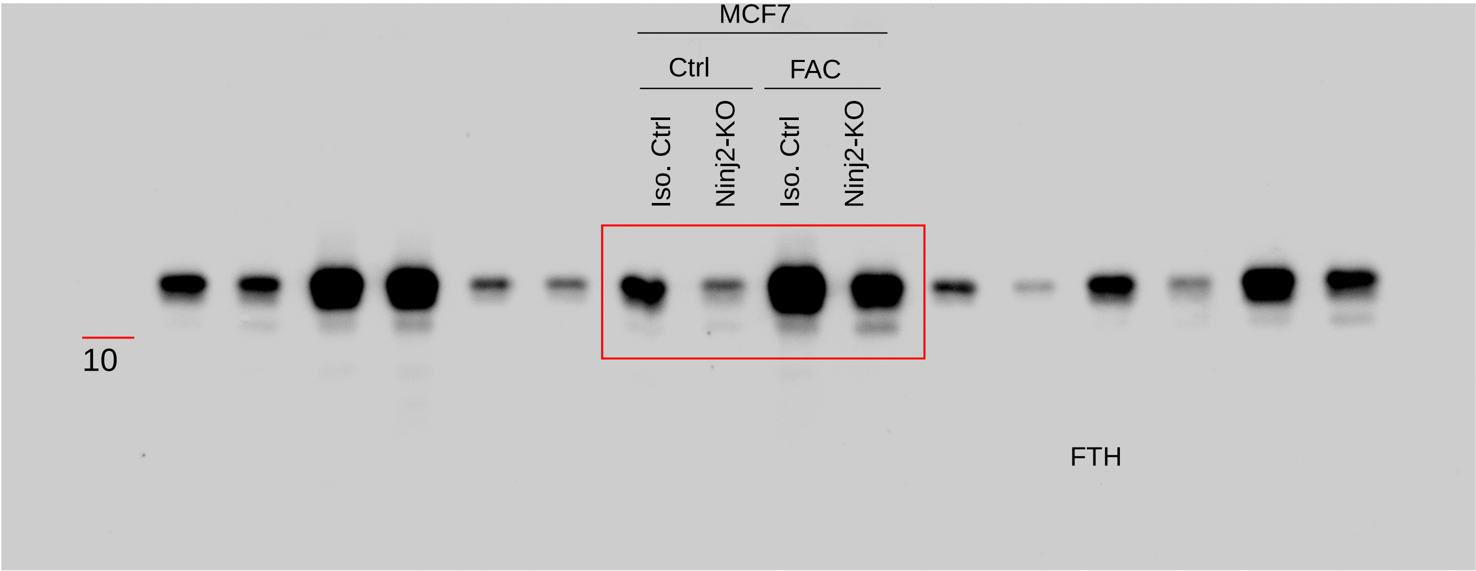

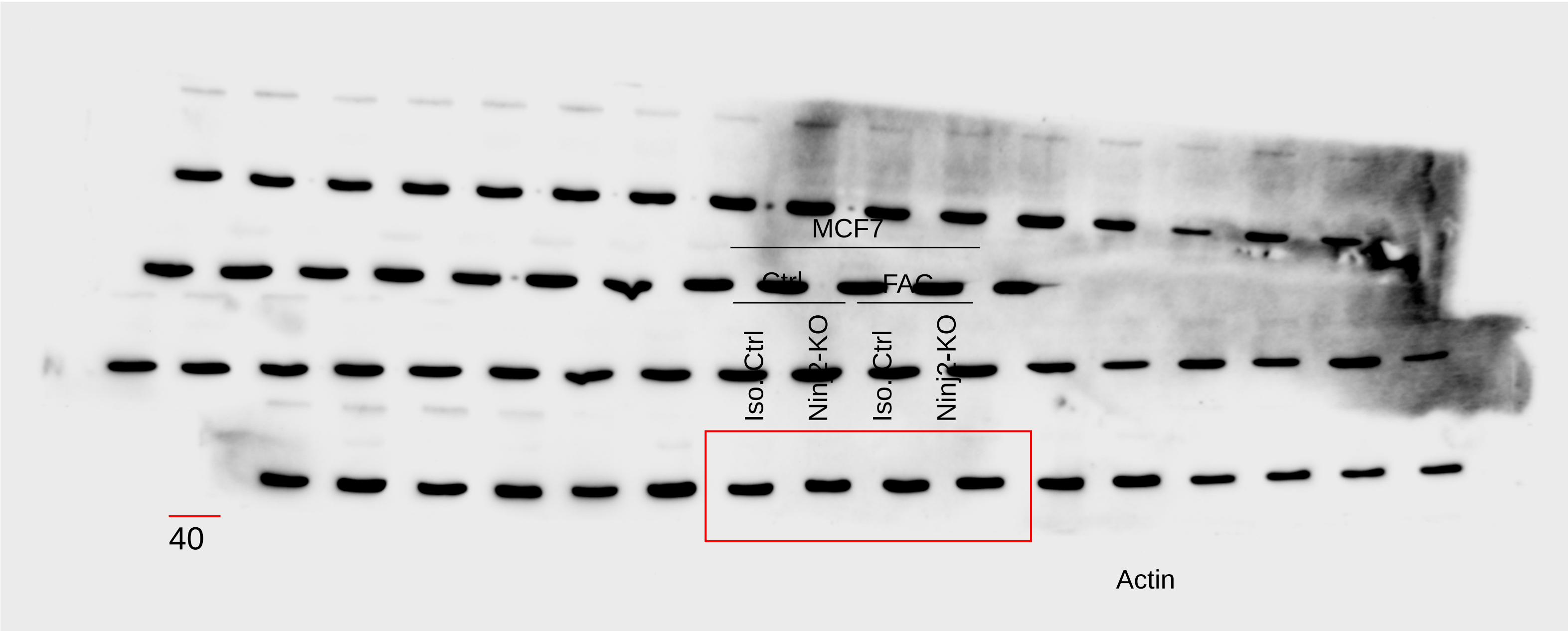

Figure 3, Source Data 1. Original membranes corresponding to Figure 3 panel F.
