# Supplementary material for: Nerve injury-induced protein 2 preserves lysosomal membrane integrity to suppress ferroptosis
Source: eLife. 2026 Sep 11;15:RP110919. doi: 10.7554/eLife.110919 (PMC13568700; doi:10.7554/eLife.110919)
Supplement: Supplementary file 10 [file elife-110919-fig4-data1.zip › Figure 4-Source data 1.pdf]

Figure 4A

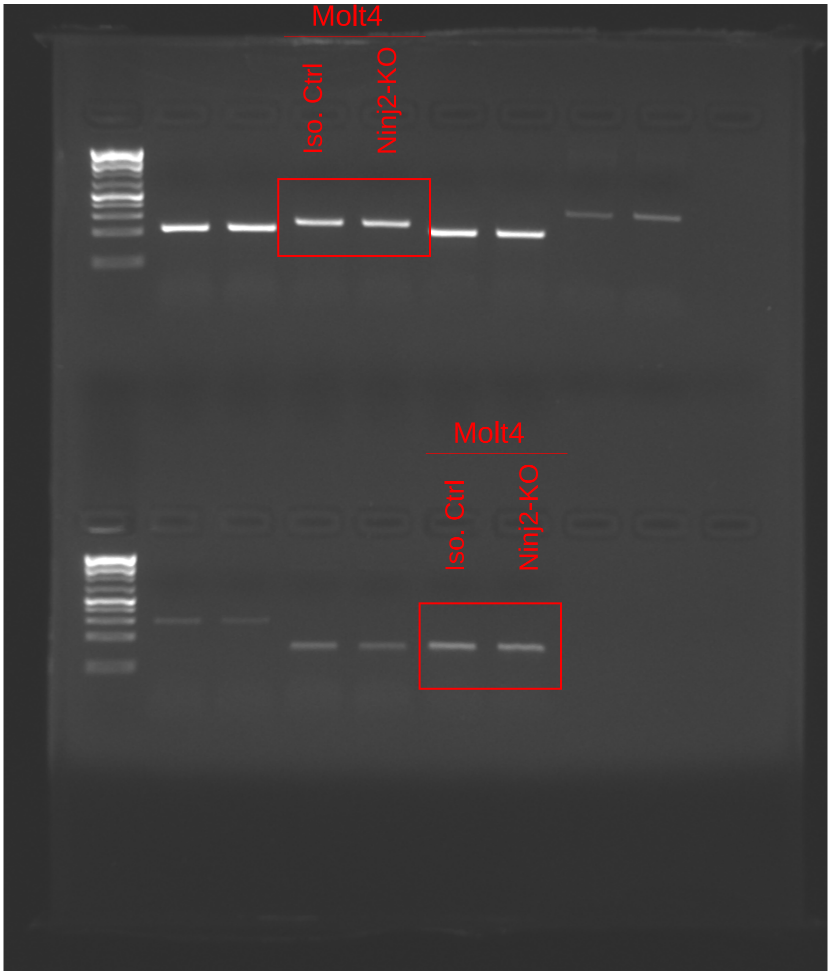

Figure 4, Source Data 1. Original gels corresponding to Figure 4, Panel A.

Figure 4B

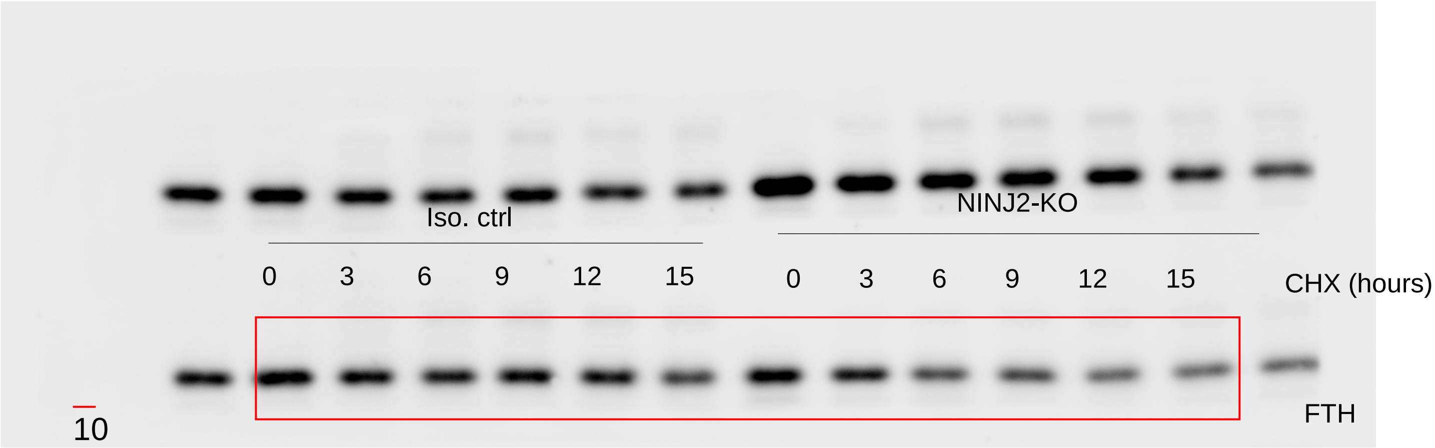

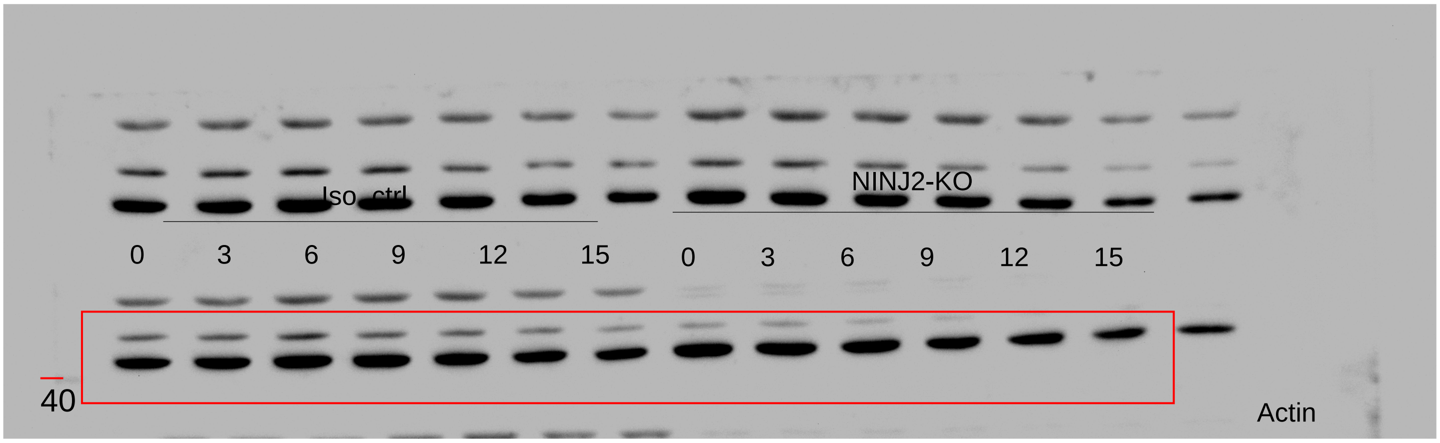

Figure 4, Source Data 1. Original Membranes corresponding to Figure 4, Panel B.

Figure 4D

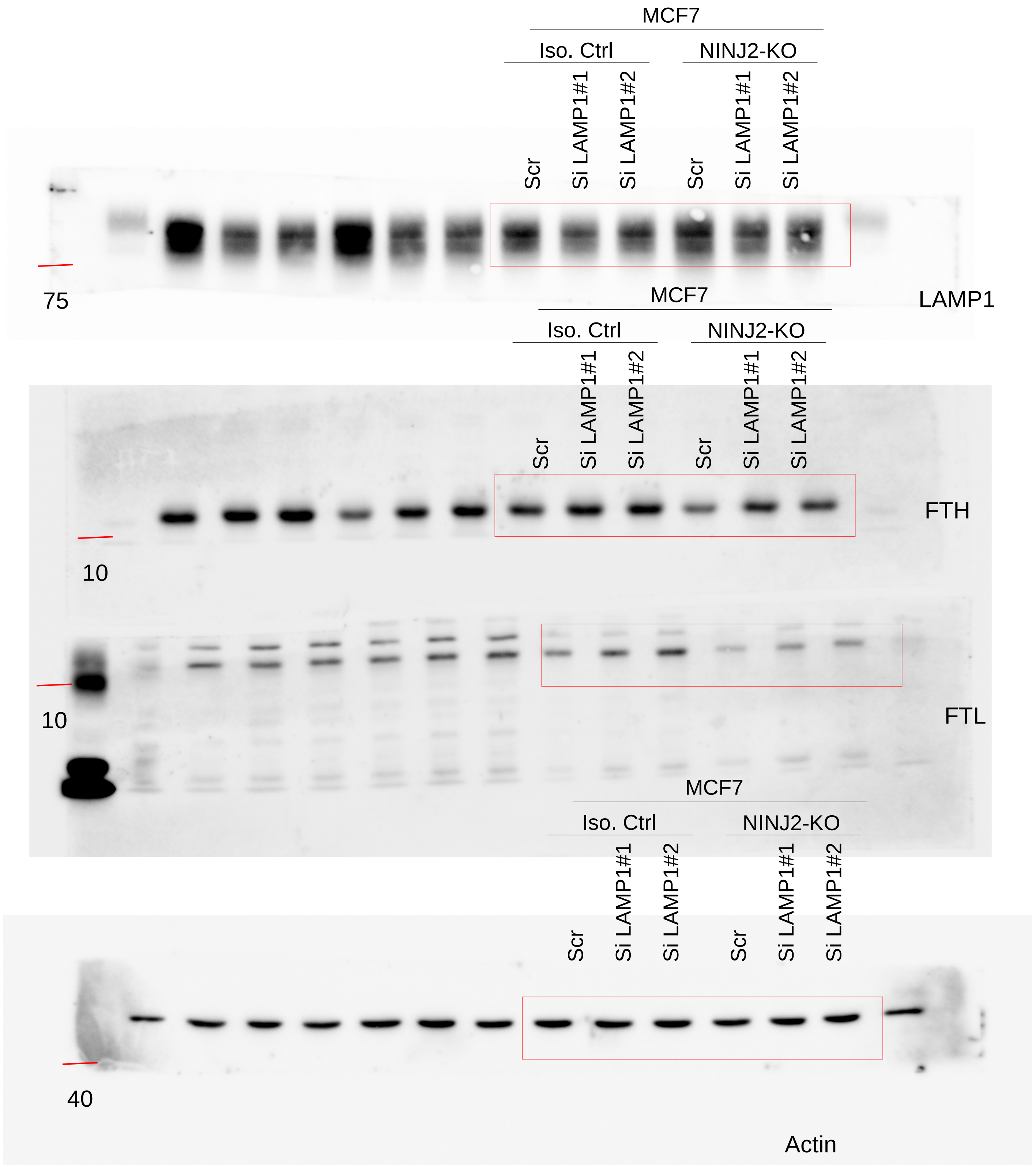

Figure 4, Source Data 1. Original Membranes corresponding to Figure 4, Panel D.
