# Supplementary material for: Nerve injury-induced protein 2 preserves lysosomal membrane integrity to suppress ferroptosis
Source: eLife. 2026 Sep 11;15:RP110919. doi: 10.7554/eLife.110919 (PMC13568700; doi:10.7554/eLife.110919)
Supplement: Supplementary file 12 [file elife-110919-fig5-data1.zip › Figure 5-Source data 1.pdf]

Figure 5A

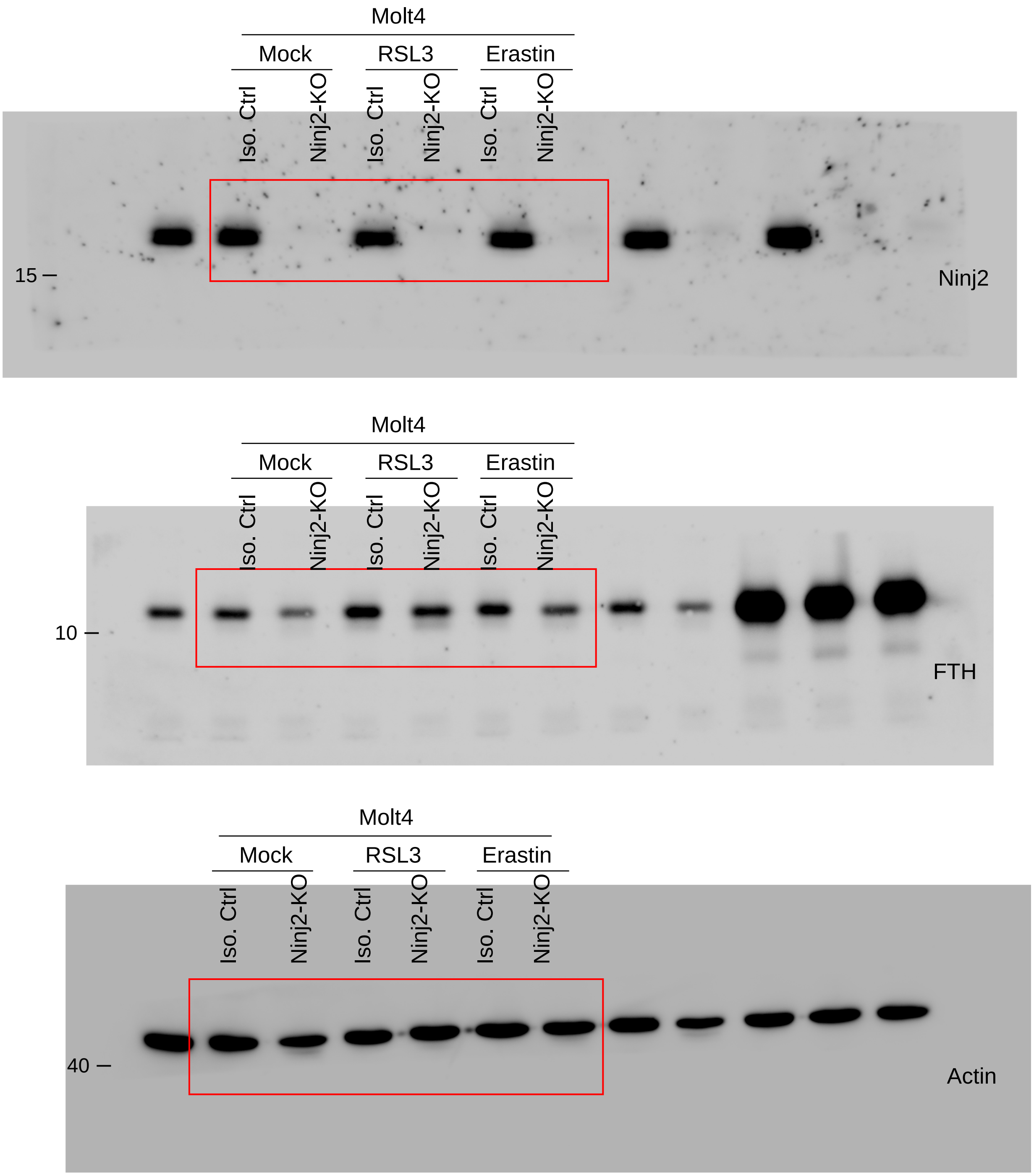

Figure 5 Source Data 1. Original membranes corresponding to Figure 5, panel A.

Figure 5B

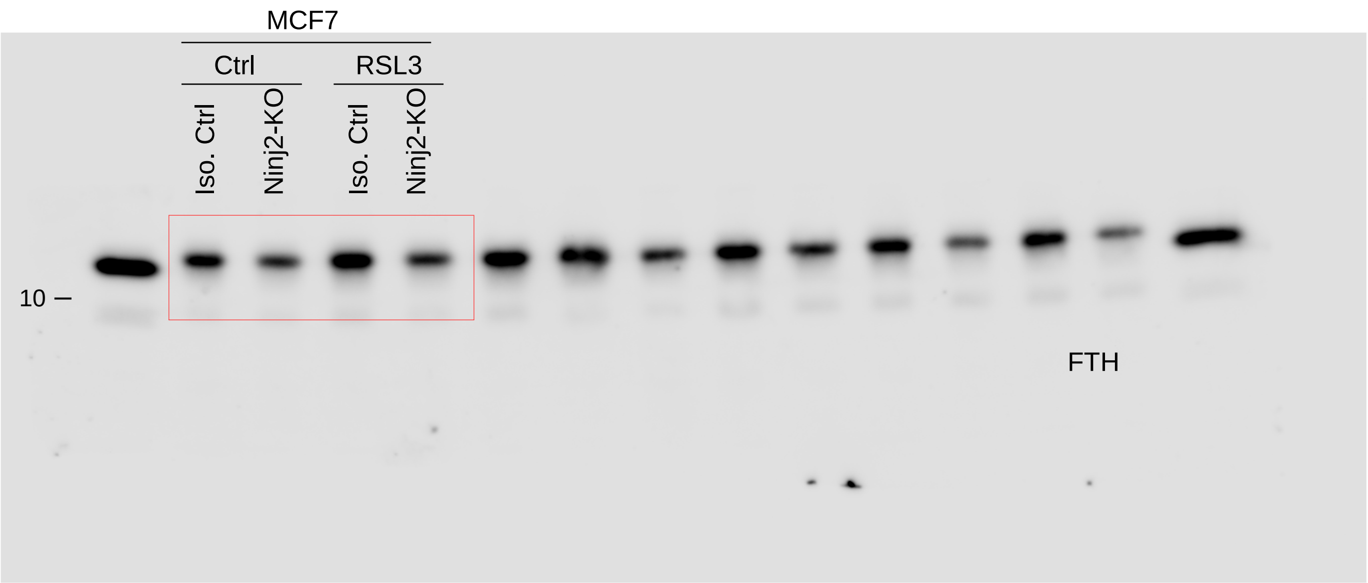

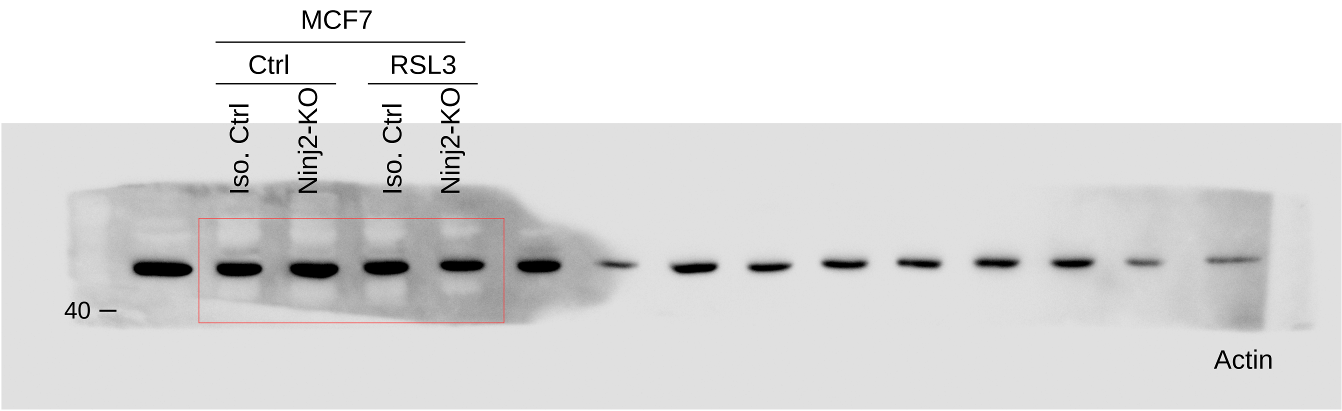

Figure 5 Source Data 1. Original membranes corresponding to Figure 5, panel B.
