# Supplementary material for: Nerve injury-induced protein 2 preserves lysosomal membrane integrity to suppress ferroptosis
Source: eLife. 2026 Sep 11;15:RP110919. doi: 10.7554/eLife.110919 (PMC13568700; doi:10.7554/eLife.110919)
Supplement: Peer review history

# Editor report: eLife Assessment

**DOI:** 10.7554/eLife.110919.3.sa0

**Authors:** Qing Zhang

**Affiliations:** University of Texas Southwestern Medical Center United States

**Keywords:** Convincing, Fundamental

This **fundamental** work uncovers an unexpected lysosomal function for NINJ2 and links it to ferroptosis and cancer biology. The evidence supporting the conclusions appears to be **convincing**. This work will be of general interest to the community of ferroptosis and cancer biology.

# Referee report: Reviewer #1 (Public review)

**DOI:** 10.7554/eLife.110919.3.sa1

Summary:

This study reports a novel and potentially impactful role for NINJ2 in maintaining lysosomal integrity and regulating cellular susceptibility to ferroptosis. The authors demonstrate that NINJ2 localizes to lysosomes and interacts with LAMP1, a key lysosomal membrane glycoprotein involved in sensing lysosomal stress. Loss of NINJ2 increases lysosomal membrane permeabilization (LMP), resulting in selective leakage of lysosomal contents, including labile iron, into the cytosol. The authors further show that NINJ2 deficiency reduces the expression of ferritin storage proteins, thereby sensitizing cells to ferroptosis induced by RSL3 and erastin. Collectively, the work proposes a mechanistic link between NINJ2-mediated control of LMP, iron homeostasis, and ferroptotic vulnerability, with potential relevance to cancer biology.

Strengths:

This study identifies a novel role for NINJ2 in regulating lysosomal integrity and ferroptosis and establishes a mechanistic link between lysosomal membrane permeabilization, iron homeostasis, and ferroptotic sensitivity, with potential translational relevance in cancer.

Weaknesses:

The results overall support the authors' conclusions and provide a plausible mechanistic framework; however, additional quantification of western blot data and further discussion of mechanistic questions would strengthen the study.

The findings are likely to have broad impact by linking lysosomal integrity to ferroptosis and iron homeostasis, both of which are relevant to cancer biology and therapeutic targeting.

Comments on revised version.

The authors have addressed all of my comments and questions. I have no further concerns.

# Referee report: Reviewer #2 (Public review)

**DOI:** 10.7554/eLife.110919.3.sa2

This manuscript, "Nerve Injury-Induced Protein 2 preserves lysosomal membrane integrity to suppress ferroptosis", identifies a previously unrecognized function of NINJ2 as a regulator of lysosomal membrane integrity and iron homeostasis, thereby suppressing ferroptosis. The authors demonstrate that NINJ2 localizes to lysosomes, interacts with LAMP1, limits lysosomal membrane permeabilization (LMP), stabilizes ferritin, and protects cells from ferroptotic cell death. They further extend these mechanistic findings to human cancer datasets, showing co overexpression and positive correlation of NINJ2 with ferritin genes in iron addicted cancers.

Overall, the study is conceptually interesting, technically solid, and integrates cell biology, iron metabolism, and ferroptosis in a coherent framework. The work expands the functional repertoire of the Ninjurin family beyond plasma membrane rupture and inflammation, which will be of interest to researchers in cell death, lysosome biology, and cancer metabolism.

Strengths:

(1) The identification of NINJ2 as a lysosome-associated protein that suppresses ferroptosis represents a meaningful advance beyond its previously described roles in inflammation, pyroptosis, and tumorigenesis.

(2) The work distinguishes NINJ2 functionally from NINJ1, reinforcing the idea that structurally related Ninjurins have divergent membrane-related roles.

(3) The study presents a logically connected pathway:

NINJ2 loss → LMP → labile iron increase → ferritin degradation → ferroptosis sensitization, which is well supported by the data.

(4) The link between LAMP1, ferritin turnover, and ferroptosis is particularly compelling and timely given recent interest in lysosomal contributions to ferroptotic signaling.

(5) The authors use confocal microscopy, proximity ligation assays, biochemical IPs, iron measurements, protein half-life analyses, ferroptosis assays, and TCGA-based analyses, providing convergent evidence for their model.

(6) Use of two distinct cell lines (MCF7 and Molt4) strengthens generalizability.

(7) The integration of cancer expression datasets linking NINJ2 with ferritin expression in hepatocellular and breast carcinomas enhances translational relevance.

(8) Assigning NINJ2 a lysosomal protective function, distinct from NINJ1-mediated plasma membrane rupture is novel.

(9) Linking NINJ2 to ferroptosis regulation via lysosomal iron handling, rather than canonical GPX4 or system Xc⁻ pathways is also novel, along with proposing a NINJ2-LAMP1-ferritin axis as a buffering mechanism against iron-driven lipid peroxidation.

(10) These insights are not incremental; they reframe how NINJ2 may function at the intersection of membrane biology, iron metabolism, and regulated cell death.

# Author comment: Author response

**DOI:** 10.7554/eLife.110919.3.sa3

**Authors:** Jin Zhang, Miranda Bustamante, Yang Shi, Ken-ichi Nakajima, Xinbin Chen

**Affiliations:** University of California, Davis Davis United States; University of California, Davis Davis United States; University of California, Davis Davis United States; University of California, Davis Davis United States; University of California, Davis Davis United States

The following is the authors’ response to the original reviews.

**Public Reviews:**

**Reviewer #1 (Public review):**

Summary:

This study reports a novel and potentially impactful role for NINJ2 in maintaining lysosomal integrity and regulating cellular susceptibility to ferroptosis. The authors demonstrate that NINJ2 localizes to lysosomes and interacts with LAMP1, a key lysosomal membrane glycoprotein involved in sensing lysosomal stress. Loss of NINJ2 increases lysosomal membrane permeabilization (LMP), resulting in selective leakage of lysosomal contents, including labile iron, into the cytosol. The authors further show that NINJ2 deficiency reduces the expression of ferritin storage proteins, thereby sensitizing cells to ferroptosis induced by RSL3 and erastin. Collectively, the work proposes a mechanistic link between NINJ2-mediated control of LMP, iron homeostasis, and ferroptotic vulnerability, with potential relevance to cancer biology.

Strengths:

This study identifies a novel role for NINJ2 in regulating lysosomal integrity and ferroptosis and establishes a mechanistic link between lysosomal membrane permeabilization, iron homeostasis, and ferroptotic sensitivity, with potential translational relevance in cancer.

Weaknesses:

(1) The results overall support the authors' conclusions and provide a plausible mechanistic framework; however, additional quantification of Western blot data and further discussion of mechanistic questions would strengthen the study.

All the western blot data have been quantified throughout the manuscript in the revised version.

(2) The findings are likely to have a broad impact by linking lysosomal integrity to ferroptosis and iron homeostasis, both of which are relevant to cancer biology and therapeutic targeting.

We thank the reviewer’s comment. We have discussed the potential implications of these findings for cancer treatment in the “Discussion” section.

**Reviewer #2 (Public review):**

This manuscript, "Nerve Injury-Induced Protein 2 preserves lysosomal membrane integrity to suppress ferroptosis", identifies a previously unrecognized function of NINJ2 as a regulator of lysosomal membrane integrity and iron homeostasis, thereby suppressing ferroptosis. The authors demonstrate that NINJ2 localizes to lysosomes, interacts with LAMP1, limits lysosomal membrane permeabilization (LMP), stabilizes ferritin, and protects cells from ferroptotic cell death. They further extend these mechanistic findings to human cancer datasets, showing cooverexpression and positive correlation of NINJ2 with ferritin genes in iron-addicted cancers.

Overall, the study is conceptually interesting, technically solid, and integrates cell biology, iron metabolism, and ferroptosis in a coherent framework. The work expands the functional repertoire of the Ninjurin family beyond plasma membrane rupture and inflammation, which will be of interest to researchers in cell death, lysosome biology, and cancer metabolism.

Strengths:

(1) The identification of NINJ2 as a lysosome-associated protein that suppresses ferroptosis represents a meaningful advance beyond its previously described roles in inflammation, pyroptosis, and tumorigenesis.

(2) The work distinguishes NINJ2 functionally from NINJ1, reinforcing the idea that structurally related Ninjurins have divergent membrane-related roles.

(3) The study presents a logically connected pathway:

NINJ2 loss → LMP → labile iron increase → ferritin degradation → ferroptosis sensitization, which is well supported by the data.

(4) The link between LAMP1, ferritin turnover, and ferroptosis is particularly compelling and timely given recent interest in lysosomal contributions to ferroptotic signaling.

(5) The authors use confocal microscopy, proximity ligation assays, biochemical IPs, iron measurements, protein half-life analyses, ferroptosis assays, and TCGA-based analyses, providing convergent evidence for their model.

(6) Use of two distinct cell lines (MCF7 and Molt4) strengthens generalizability.

(7) The integration of cancer expression datasets linking NINJ2 with ferritin expression in hepatocellular and breast carcinomas enhances translational relevance.

(8) Assigning NINJ2 a lysosomal protective function, distinct from NINJ1-mediated plasma membrane rupture, is novel.

(9) Linking NINJ2 to ferroptosis regulation via lysosomal iron handling, rather than canonical GPX4 or system Xc- pathways, is also novel, along with proposing a NINJ2-LAMP1-ferritin axis as a buffering mechanism against iron-driven lipid peroxidation.

(10) These insights are not incremental; they reframe how NINJ2 may function at the intersection of membrane biology, iron metabolism, and regulated cell death.

Areas for improvement:

While the study is strong, several issues should be addressed for mechanistic depth and general relevance.

(1) Although NINJ2 is shown to interact with LAMP1 and LAMP1 knockdown rescues ferritin levels, it remains unclear whether the NINJ2-LAMP1 interaction is required for lysosomal protection. The authors could: (a) Map the NINJ2 domain required for LAMP1 interaction and test whether an interaction-deficient mutant fails to protect against LMP and ferroptosis. (b) Rescue NINJ2 KO cells with wild-type versus mutant NINJ2 to establish causality.

We thank the reviewer’s comments. Ongoing work in our laboratory is focused on elucidating the molecular mechanism by which the NINJ2-LAMP1 interaction regulates lysosomal membrane integrity and ferroptosis, and we anticipate reporting these findings in a future publication.

(2) The conclusion that NINJ2 suppresses ferroptosis relies primarily on RSL3 and Erastin sensitivity. A direct assessment of ferroptosis would hence the study, such as:

(a) Include ferroptosis rescue experiments using ferrostatin 1 or liproxstatin 1.

(b) Assess lipid peroxidation directly (e.g., C11 BODIPY staining) to strengthen the ferroptosis claim.

We thank the reviewer for this thoughtful comment. We agree that ferrostatin-1 or liproxstatin-1 rescue experiments, together with direct analysis of lipid peroxidation, would provide complementary evidence for ferroptosis. We will incorporate these additional experiments in future studies to further strengthen the mechanistic basis of our findings.

(3) The manuscript discusses lysosomal ferritin degradation but does not directly examine NCOA4, a central mediator of ferritinophagy. It would be good to: (a) Test whether NCOA4 knockdown rescues ferritin loss and ferroptosis sensitivity in NINJ2 KO cells. (b) This would clarify whether NINJ2 acts upstream of canonical ferritinophagy pathways or via an alternative mechanism.

We appreciate the reviewer's thoughtful suggestion. Defining the contribution of NCOA4 to NINJ2-mediated ferritin degradation is an important question that could further clarify the underlying mechanism. Addressing this issue will require a comprehensive set of additional experiments, which will be addressed in the future studies.

(4) The study is entirely cell-based, despite references to inflammatory and tumor phenotypes in Ninj2-deficient mice. While not strictly required, even limited in vivo validation (e.g., ferroptosis markers or iron accumulation in existing Ninj2 KO tissues) would substantially strengthen the manuscript.

We thank the reviewer for this insightful suggestion. We agree that *in vivo* validation of ferroptosis markers and iron accumulation in Ninj2-deficient tissues would further strengthen our conclusions. However, these experiments will require substantial additional investigation, which will be pursued in the future studies.

(5) Finally, most imaging data (e.g., Galectin 3/LAMP1 colocalization, PLA signals) and immunoblot data are presented qualitatively. The authors should provide the qualifications of Western blots and other measurements.

All the western blot data have been quantified throughout the manuscript in the revised version.

**Recommendations for the authors:**

**Reviewer #1 (Recommendations for the authors):**

(1) What mechanisms might underlie the regulation of LAMP1 transcript levels by NINJ2?

A clear mechanism by which NINJ2 regulates LAMP1 transcripts has not been elucidated and warrants further investigation. Nevertheless, several possibilities can be considered. First, LAMP1 transcription is known to be regulated by TFEB (transcription factor EB), a master regulator of the lysosomal–autophagy pathway. Upon lysosomal membrane permeabilization (LMP), TFEB translocates to the nucleus and activates a broad set of lysosome-related genes, including LAMP1. Notably, phosphorylation of TFEB by mTORC1 at Ser211 inhibits its activity by preventing nuclear translocation. Thus, it would be of interest to determine whether NINJ2 modulates TFEB phosphorylation status and subcellular localization. In addition, the tumour suppressor p53 has been reported to engage in complex crosstalk with TFEB in regulating basal autophagy. Interestingly, p53 expression is increased in NINJ2-KO cells. It is therefore plausible that NINJ2 regulates TFEB activity through p53, or alternatively modulates the p53–TFEB signaling axis more broadly to maintain lysosomal integrity.

(2) Does Ninjurin1 play a similar role in regulating lysosomal membrane permeabilization (LMP)?

At this moment, it remains unclear whether NINJ1 plays a role similar to that of NINJ2 in regulating LMP. In fact, our previous studies demonstrated that NINJ2 physically interacts with NINJ1 and may antagonize NINJ1-mediated pyroptosis. Furthermore, NINJ1 has recently been reported to promote ferroptosis by interacting with the xCT cystine/glutamate antiporter (PMID: 38464226), a function that contrasts with the protective role of NINJ2 against ferroptosis identified in the present study. These findings suggest that NINJ1 and NINJ2 may have distinct, or even opposing, functions in regulating cell death pathways. Nevertheless, further studies are required to determine whether NINJ1 also participates in the regulation of LMP and to define its relationship with NINJ2 in maintaining lysosomal membrane integrity.

(3) What are the potential clinical implications of these findings, particularly in the context of cancer progression or therapeutic targeting?

Targeting NINJ2 may have important clinical implications in cancer therapy. Given its role in maintaining lysosomal membrane integrity, inhibition or loss of NINJ2 could promote lysosomal membrane permeabilization (LMP), thereby sensitizing cancer cells to ferroptosis through increased intracellular labile iron accumulation and disruption of redox homeostasis, ultimately enhancing tumor cell killing. As such, NINJ2 inhibition may represent a strategy to selectively destabilize lysosomal function in cancer cells and improve responsiveness to ferroptosis-inducing agents or other combination therapies that exploit oxidative stress vulnerabilities. Indeed, we previously developed a peptide that targets NINJ2. Whether this NINJ2-targeting peptide can sensitize cancer cells to ferroptosis therefore warrants further investigation.

(4) The authors should provide quantification of all Western blot data throughout the manuscript to enhance data robustness and reproducibility.

All the western blot data have been quantified throughout the manuscript in the revised version.

**Reviewer #2 (Recommendations for the authors):**

(1) Controls for knockdown efficiency of NINJ2 (Figure 2D) should be shown.

NINJ2-KO MCF7 cells were generated previously and published in the article (PMID: 38325550) along with sequencing confirmation.

(2) In Figure 2A legends, the concentration of LLOMe is 1mM or 1µM - need to be clarified?

The concentration for LLOME is 1µM. This typo has been corrected.

(3) In the figure legends section, "Figure 4" is missing.

We thank the reviewer’s comment. Figure 4 has been added to the Figure legends.

(4) Some description of NINJ2 ko cells generation should be included in the materials section.

In the Materials and Methods section, we briefly described how these cell lines were generated and cited the original publication (PMID: 38325550)

(5) The manuscript would benefit from a schematic model figure summarizing the proposed NINJ2-LAMP1-iron-ferroptosis axis.

In the revised manuscript, we provided a model to elucidate the role of NINJ2 in modulating lysosomal membrane integrity and iron homeostasis.

(6) Some sections of the Introduction are lengthy and could be streamlined to focus more directly on lysosomes and ferroptosis.

We have streamlined the introduction.

(7) Statistical methods should clarify whether data meet assumptions for Student's t-test and whether multiple comparisons were corrected where applicable.

Statistical analysis has been added to the figure legends when applicable.
